# Barriers to Diabetes Self-Management in a Subset of New Zealand Adults with Type 2 Diabetes and Poor Glycaemic Control

**DOI:** 10.1155/2021/5531146

**Published:** 2021-05-27

**Authors:** Lynne Chepulis, Brittany Morison, Shemana Cassim, Kimberley Norman, Rawiri Keenan, Ryan Paul, Ross Lawrenson

**Affiliations:** ^1^Medical Research Centre, University of Waikato, Hamilton, New Zealand; ^2^Waikato District Health Board, Waikato, Hamilton, New Zealand

## Abstract

**Background:**

Despite the fact that there is an increasingly effective armoury of medications to treat diabetes, many people continue to have substantially elevated blood glucose levels. The purpose of this study was to explore what the barriers to diabetes management are in a cohort of people with diabetes and poor glycaemic control.

**Methods:**

Qualitative semistructured interviews were carried out with 10 people with diabetes who had known diabetes and a recent HbA1c of >11.3% (100 mmol/mol) to explore their experiences of barriers to diabetes self-management and glycaemic control.

**Results:**

Barriers to diabetes management were based around two key themes: biopsychosocial factors and knowledge about diabetes. Specifically, financial concerns, social stigma, medication side effects, and cognitive impairment due to hyperglycaemia were commonly reported as barriers to medication use. Other barriers included a lack of knowledge about their own condition, poor relationships with healthcare professionals, and a lack of relevant resources to support diet and weight loss.

**Conclusion:**

People with diabetes with poor glycaemic control experience many of the same barriers as those reported elsewhere, but also experience issues specifically related to their severe hyperglycaemia. Management of diabetes could be improved via the increased use of patient education and availability of locally relevant resources.

## 1. Introduction

Type 2 diabetes mellitus (T2DM) is a chronic condition that currently affects approximately 7% of New Zealand's population, including a disproportionate number of Māori, the indigenous people of New Zealand [1, 2]. Importantly, as in other countries, the prevalence of T2DM in New Zealand is increasing year on year due the close association with obesity [3]. Further, a greater number of younger people are now being affected [4].

Medically, the goals of T2DM management are to optimally manage glycaemia (measured, in part, by glycated haemoglobin (HbA1c) measurements) and other cardiovascular risk factors such as hypertension and dyslipidaemia, with the aim to prevent, delay, or slow the progression of microvascular and macrovascular complications [5, 6]. However, despite the fact that there have been significant improvements in the availability of medications to treat diabetes, many people with T2DM continue to have poor glycaemic control. Indeed, in a recent review of more than 3500 people with T2DM in New Zealand, more than half had a most recent HbA1c of >55 mol/mol (7.2%) [7]. This is a substantially higher proportion of patients than in most European countries [8] and the USA [9].

The management of T2DM is multifaceted, including patient education, medication, and lifestyle changes [10, 11]. In primary care in New Zealand and elsewhere, management of T2DM focuses on promotion of a healthy lifestyle, regular monitoring of clinical measures, use of medication, and specialist referral as required [11]. In New Zealand, general practitioners (GPs) are expected to offer all people with diabetes an annual review of their risk of diabetes-related complications, though recent data suggests that only 50-60% of all people with diabetes complete this annually [7].

However, despite the implementation of these health system strategies, many studies have identified that patient-level barriers can significantly impact on glycaemic control and diabetes management [12–16]. To date, these issues have not been investigated in detail in New Zealand though in 1998 a multiethnic qualitative study in South Auckland looked to identify barriers to diabetes care from both the individual and the healthcare provider (HCP) perspective. This study identified that barriers spanned many different themes, including the need for translated educational material, psychological barriers, and physical and social barriers to care [17]. Similarly, in 2007, in a cohort of people with T2DM, psychological factors (strictness of medication regimen and motivation) were ranked as being important barriers to care by both individuals and HCPs [18]. Further, in 2013, a small study with rural people with diabetes reiterated the fact that barriers to glycaemic control are varied and many, but also reported that these can include a lack of medication adherence and a lack of understanding by the clinician of the participants' fears, beliefs, and expectations around their diagnosis and condition management [19]. Importantly, however, none of these three studies reported on the HbA1c measurements at the time of the interview and it is impossible to determine how barriers may differ between those who have good versus bad glycaemic control. Indeed, despite the fact that structured diabetes education is becoming more recognised as a critical component of diabetes management [20–22], international studies continue to report that people with very poor glycaemic control and/or poor diabetes management may present with unique barriers and challenges [23]. These include becoming easily frustrated in situations such as delayed or inappropriate use of insulin therapy [24, 25] and significant deficits in diabetes knowledge [23], indicating that many could benefit from more individualised management.

To date, no study has reported on the barriers to glycaemic control as they pertain to New Zealand people with diabetes with excessively high HbA1c levels. Thus, this study was designed to provide a recent evaluation of the barriers to diabetes care and glycaemic control specifically in those with T2DM who have very poor glycaemic control.

## 2. Methods

### 2.1. Setting

This study was conducted in the Waikato region of New Zealand, as part of a larger Waikato-based “Diabetes in Primary Healthcare Study.” Participants for this qualitative study were recruited from two general practices: one based in an urban locality and the other in a rural locality. These two practices were chosen because they have previously agreed to participate as a research practice.

### 2.2. Participants and Recruitment

A subset of 100 people with T2DM with poor glycaemic control (HbA1c > 11.3%; 100 mmol/mol) was selected at random from the two general practices (50 from each) and invited via a letter to participate in this qualitative study. Participants had no prior knowledge of the researchers or the project prior to being contacted, though all were provided with a participant information sheet describing the research team and the nature of the project. Of the 100 letters sent, only ten participants responded to our invitation, and thus, all were included and interviewed for this study. All participants were given the option to have family present at the interview. Three participants completed the interview with their partner/spouse present, and any relevant comments from these people were noted. Overall, participants comprised five Māori, four New Zealand European (NZE), and one Asian adult. Six of the participants were female, and all were aged ranged between 26 and 75 years. Three participants were enrolled with the rural practice, and seven were enrolled with the urban practice.

The study took place between July and August 2019. Participants were asked to discuss their experiences as a person with T2DM and their experiences of barriers to quality diabetes care. Semistructured interviews were carried out with each participant by BM, a trained female researcher using an interview guide prepared specifically for this study. Each interview was approximately 30-60 minutes in duration and took place at either the participants' homes or a local café. Family was allowed to be present during the interview.

### 2.3. Analysis

Participant interviews were recorded as field notes and via an audio recorder. Audio recordings were transcribed and anonymized. Pseudonyms have also been used to ensure the anonymity of participants. Transcripts and field notes were thematically analysed [26]. Analysis was carried out by two experienced researchers (BM and KM) independently first and then together to ensure a rigorous analysis process. Final analysis was overseen by a third researcher (SC), where general themes and subthemes were discussed, constructed, reviewed, and refined. Participants were given the option to see their interview transcripts but all declined this opportunity. All were provided with a summary of the overall findings.

## 3. Results

Data analysis identified two overarching themes contributing to poor glycaemic control: “biopsychosocial barriers” and “knowledge about diabetes”

### 3.1. Biopsychosocial Barriers

Participants indicated that having T2DM and the continued need to control their glycaemic levels had a significant impact on various other areas of their lives (financial, social, physical, and cognitive). Such affects tended to occur in a negative feedback loop: while the condition affected these various aspects of peoples' lives, the financial, social, physical, and cognitive factors also then acted as barriers to achieving and maintaining optimal glycaemic control.

#### 3.1.1. Financial

Participants highlighted significant financial costs associated with living with T2DM. For instance, six participants pointed out that the mounting costs of medication (particularly insulin), mandatory healthcare provider (HCP) visits, alongside the need to take time off work and transport costs were a day-to-day reality of living with T2DM. Such accumulating costs then made it difficult to continue maintaining/achieving optimal glycaemic control:

*“It starts hitting your pocket … that's how bad it got, I was spending about 65-100 bucks just on medication every three weeks.” (Simon, male, 42, urban)*

*“It kind of added up, and I didn't have enough money to pay for it and I remember standing there thinking should I ask the girl to put it on sort of an account for me, but you know my pride got the better of me and I just said can you just put it to the side and I'll return. I returned like two weeks later because I was being paid fortnightly and so I had to suffer ‘cause I wasn't getting any medication at that time.” (Sean, male, 37, urban)*

#### 3.1.2. Social

The majority of participants also reported that stigma/social judgements received from others in public, or from family members when taking their medication, became a barrier to self-management of T2DM. Insulin injections were particularly disliked, primarily because of the impact that this had on other family members. Debbie reported how her daughter had made negative comments about her injecting insulin:

“*I would be getting the [insulin] pen out, and my daughter walks in ‘ewww don't mum, not in my bloody kitchen you don't'.” (Debbie, female, 58, urban)*

Consequently, participants tended to avoid taking their medication in order to shield their family members from the entire seemingly unpleasant process. For instance, Simon reported that he did not think it was socially acceptable to be injecting himself with insulin in front of his young son in case his son copied him and hurt himself with the needles:

*“I don't want to do injections, and that's one of the reasons I stopped for quite a while -because of my son being so young. [I] didn't want him to see me doing that and think [that] it was normal….so I stopped taking it for like six months.” (Simon, male, 42, urban)*

#### 3.1.3. Physical

Five participants also indicated that the side effects of T2DM medication served as a barrier to glycaemic control. As a result, these participants refused to take their medication, actively changed their medication, or found alternate remedies that fit with their individual lifestyle. Phil admitted that he stopped his medication because it affected his work:

*“The medication [metformin] yes, I did change it, I think it's effecting me now…. I get diarrhoea, so I try not to take it while I'm working cause I'm on mobile patrol so it's not ideal.” (Phil, male, 42, rural)*

Sean, on the other hand, convinced his HCP to change his medication due to its adverse side effects:

*“I didn't like the Metformin because I just felt like it was honestly wrecking my insides and the doctor understood … and so I pressured the doctor to push me onto the insulin.” (Sean, male, 37, urban)*

Given the negative side effects of the medication, participants actively sought out alternative methods for controlling their blood glucose levels, including experimenting with the effects on their body after prolonged periods of not taking their diabetic medication and using cinnamon or a particular diet plan (e.g., Keto) to help control their diabetes. However, the side effects of these alternatives seemed to be worse that the medication itself, resulting in substantial increases in HbA1c levels and reduced self-reported quality of life (including teasing from friends and feelings of disappointment). Therefore, both participants later returned to medication, upon advice from their general practitioner.

“*I've looked at other methods of dealing with it [T2DM] like having cinnamon and stuff, because cinnamon is apparently meant to be really good for diabetes. So I was just trying to take it [cinnamon], instead of having all these pills.” (Simon, male, 42, urban)*

#### 3.1.4. Cognitive and Psychological

Additionally, participants reported concerns about their diabetes having a negative effect on their cognitive health, which impacted on their ability to effectively manage their condition. For instance, some participants thought that their diabetes influenced their mental health, indicating that the condition prevented them *“from being able to think straight” (Laura, female, 49, urban)*. Importantly, this lack of mental clarity was observed to be worse during periods of poorer glycaemic control, which then further impacted on a number of factors that made self-management of T2DM difficult. For instance, participants commonly reported the inability to remember to take their medication:

*“It's not very nice when you can't think because your brain fog.” (Laura, female, 49, urban)*

*“Cause man, half the time I don't remember to take my pills.” (Simon, male, 42, urban)*

Consequently, several participants devised strategies to help them remember. However, even this was impacted by memory loss due to poor glycaemic control:

*“Sometimes I have forgotten, like today. Normally I am in a habit of putting the [insulin] pen on the table which I forgot to do this morning.” (Debbie, female, 58, urban)*

Some participants also recognised that they were not consciously aware of the “brain fog” while their HbA1c levels were excessively high, though they often reported the ability to “think clearer” when their HbA1c levels were lower. In contrast, others did recognise symptoms of poor mental health functioning during the time when their diabetes was not being managed well. These participants all indicated that it was not until they made significant lifestyle and medical changes which resulted in good glycaemic control that they understood the severity of their cognitive impairment.

Overall, participants reported that they perceived their diabetes as unfixable, feeling powerless in their management, and that they are being “punished” and forced to live with the condition indefinitely. Commonly, the condition was described as being a burden, overwhelming, and a liability. Two participants also wished that the condition had been better explained to them by their HCP or that they had received more appropriate resources about T2DM while they were in the earlier prediabetic state as this would have played a significant role in their health management. Accordingly, the ability to appropriately manage T2DM was also affected by a person's level of knowledge and understanding about the condition, alongside the information provided to them by their HCP and other publicly accessible resources.

### 3.2. Knowledge about Diabetes

Knowledge and awareness about T2DM in terms of disease biology, its implications for ongoing health, and how to manage the condition are vital for understanding the need to maintain good glycaemic control. However, based on the participant accounts from this study, it is clear that many people with diabetes may not have a clear understanding on what T2DM is, nor how it should be effectively managed. The lack of knowledge about diabetes is related to three key factors: participant factors, HCP factors, and resources and other available information. “Participant factors” include a lack of knowledge about diabetes that led to a mismanagement of their condition, “HCP factors” include the various instances where HCPs failed to provide patients with adequate information knowledge on how to appropriately manage their T2DM, and “resources and other available information” include the limitations relating to the other resources available to patients about T2DM.

#### 3.2.1. Participant Factors

Six participants reported not having a full understanding of their T2DM, and this was identified as a significant barrier to glycaemic control. Weight management is considered to be an important factor that affects T2DM control, though participants reported being unequipped to know how to lose weight with diabetes. Laura, for example, alluded to feeling trapped with her diabetes because when she tried to do something to reduce her weight she would often experience a hypoglycaemic episode:

*“Every time I would go on a weight loss programme my sugars would crash and I'd end up in hospital because they were at 1.0 [mol/mol]. Then they'd shove sugar down my throat.” (Laura, female, 49, urban)*

Participants also reported experiencing hypo- and hyperglycaemic episodes which influenced their diabetes control behaviour of trying to cut down on their sugar in order to lower their blood glucose levels:

*“I have had one slip up where I had took my insulin and stuff, went for a walk and I collapsed on the side of the road.” (Simon, male, 42, urban)*

As a result, Simon lost his trust in his dietary plan and was reluctant to cut down on his sugar intake and alluded to his body being different to others and needing sugar to feel normal. Two other participants reported similar stories where there was a lack of knowledge about how to manipulate their insulin dose when working longer shift hours and not being able to consume food at normal times.

The majority of participants with diabetes also had a very limited understanding of HbA1c (despite all having a recent measurement of >100 mmol), how T2DM affects the body, and how their diabetic medication works. One participant acknowledged this misunderstanding, yet simultaneously indicates that she still does not have clear knowledge about her medication:

*“I always thought that metformin was supposed to bring my sugar level down. I only just found out that it's not…they're to keep your valves or your arteries open or something like that.” (Laura, female, 49, urban)*

Other participants reported that their diabetes was poorly controlled because they did not understand the extent of severity of their condition and several preferred to ignore the direct impact that T2DM was having on their body. Penny, for instance, only accepted years later that she had sustained physical damage to both her feet and eyes that was caused by diabetes:

*“The bottom of my leg is discoloured and [it] has been for a number of years. It was kind of like the first warning that I had diabetes, it was the first sign and I knew that, and I just chose to ignore it…. I went to have my eyes checked this year for the first time, and they showed me the damage that was done to my eyes that won't be healed again.” (Penny, female, 64, urban)*

The importance of consuming food with medication and insulin levels needing to be adjusted in response to the quantity of carbohydrate eaten were both factors that several participants reported that they were unaware of and did not understand. Accordingly, this lack of understanding had negative impacts on these participants' ability to effectively manage their diabetes and therefore acted as a key barrier.

#### 3.2.2. Healthcare Professional Factors

Participants reported negative experiences with HCPs when dealing with their T2DM, and this was also identified as a barrier to them gaining a better understanding of their condition and thus served as a barrier to glycaemic control. The majority reported issues that included a lack of cultural awareness, lack of appropriate communication, mistrust in the HCP, and perceived insufficient information being provided by the HCP. Four participants found that their HCP was not overly helpful:

*“There wasn't much help from the health professionals [nurses]…… their answers when I did talk to them were…….* yes they were kind of, quite snarky.*” (Phil, male, 42, NZE, rural)*

*“I've learnt to favour the GPs that will support you now and ignore the ones who don't.” (Simon, male, 42, urban)*

Some participants with diabetes did receive support in the form of pamphlets or were directed to online resources on diabetes from some GPs and other practice staff such as nurses. Others sought these resources themselves. However, all found that the resources available for people with diabetes in New Zealand were not directly relevant and thus were unhelpful to them.

#### 3.2.3. Resources and Other Available Information

A lack of availability and access to relevant resources was a major theme that participants reported as impacting on their diabetes management experience. Participants reported that the information they received from their HCPs was outdated and not culturally appropriate:

*“They gave me a diet book … [there's] a non-cultural perspective, but I think they were also outdated which makes them not that relevant.” (Penny, female, 64, urban)*

Some participants attempted to seek out their own health education through online educational tools, to gain a better understanding of diabetes. However, these were also not relevant to the New Zealand context, given they were mostly tailored to the American market:

*“There is a YouTube channel, I've been watching then for a while, but she's American so it's hard to follow, they've got different stuff over there.” (Rose, female, 25, urban)*

Others resorted to seeking out secondary specialists in an effort to gain more advice. However, this option was not available for those living in rural localities:

*“I went, tried to suss out a nutritionist or something like that, to get ideas and things, but there's no one around here.” (Phil, male, 42, rural)*

Finally, participants expressed an interest in having access to ideas and resources on how they could make diabetic dietary changes through cooking and food that suited their lifestyle in the New Zealand context. Such resources could serve as another tool to boosting a person's confidence and motivation to actively control and maintain their glycaemic levels [27].

## 4. Discussion

As this study shows, people with T2DM with very poor glycaemic control may have a range of barriers that lead to reduced self-management of their condition. Many of these barriers are similar to those reported in other studies in New Zealand [17–19], despite the fact that these earlier studies likely included people with diabetes with varying degrees of glycaemic control. Participants in our study, for example, also reported shame associated with insulin injections, significant fear and altered behaviour to prevent hypoglycaemia, and lack of condition awareness, suggesting that these factors may be relatively common in people with diabetes (particularly insulin users), irrespective of a person's HbA1c levels. However, our study also demonstrates that poorly controlled diabetes left participants feeling cognitively impaired or with a state of “brain fog” that made it difficult to remember and complete even simple tasks (including taking medication). Indeed, diabetes is known to associate with a greater cognitive decline compared with no diabetes, and impairment has been shown to be worse in those with poorly controlled condition [28, 29]. Although we have not directly measured cognitive performance, the findings of our study support previous research demonstrating that cognitive impairment may lead to worse diabetes management [30]. Several participants indicated that they forgot to take their medication because they were unable to think clearly, likely as a result of the severe hyperglycaemia. However, this appears to create a negative feedback loop in which the reduced medication adherence can then lead to even worse glycaemia—this may lead to even more “brain fog” and reduced diabetes self-management [30, 31]. This lack of mental clarity needs to be recognised and understood in people with very poor glycaemic control, ideally with support provided to counter the depression, emotional distress, and forgetfulness that can result [32]. Further, in those who have significantly elevated HbA1c levels, strategies for improving medication adherence should be emphasized, as improved diabetes control has conversely been shown to create a positive feedback loop and improve cognitive performance [33].

Our study also highlighted two additional significant barriers that were specific to glycaemic control in New Zealand: the mounting person-level costs associated with diabetes management and the limited resources available to individuals and whānau (family) about their condition. Focusing on the first point, despite New Zealand's public health system being at a relatively high standard in terms of access to healthcare and medication, compared to other countries in the OECD, participants in our study highlighted that financial cost was a significant barrier to diabetes management despite the fact that the day-to-day management costs are minimal compared with other countries. For example, people with diabetes in New Zealand do have access to free and/or highly subsidised insulin, hypoglycaemic agents, glucose strips, etc., though this does require a visit to a general practitioner and a minimal prescription charge. People who are regularly attending primary care also have access to high user healthcare cards, which entitles them to reduced costs for doctors' visits and prescriptions if they meet certain criteria; however, the use of these cards has not been evaluated, and it is unknown whether the lower healthcare costs associated with these cards are passed onto people with diabetes as often as they could be. We suggest, therefore, that it would be beneficial to better understand how financial difficulties impact on T2DM management in New Zealand (and elsewhere), as other studies have reported significant improvements for primary care patients with diabetes after initiating financial support programs [34, 35].

Secondly, the limited resources provided to people with T2DM in New Zealand may also be a direct barrier to glycaemic control and self-management of the condition. Despite the availability of diabetes education resources [36–39], participants stated that their HCPs often failed to provide adequate or up-to-date and thus relevant information. Those who were proactive with trying to improve their health and diet often struggled to find information that was relevant to them. Several participants indicated, for example, that they actively sought resources on the Internet to learn more about diabetes and the foods that they should and should not eat. However, in a New Zealand context, the information available on the Internet appears to be lacking, particularly around culturally appropriate and responsive resources that are applicable to Māori. For instance, while several participants looked to YouTube for videos on healthy eating with diabetes, the videos were primarily American and entirely out of context for New Zealand people with diabetes. This is concerning, particularly as research from the last 25 years consistently showed that there are local ethnic and geographical differences in diabetes knowledge, education, and condition predisposition [21, 40–42]. As highlighted by Lambrinou and colleagues [43], patient education and support for self-management are fundamental to diabetes care. As such, steps need to be taken to provide better access to culturally appropriate information on diabetes and its treatment for people in New Zealand, which can help them manage their condition well and also prevent barriers such as misconception and stigma/social judgement.

### 4.1. Strengths and Limitations

The strength of this study lies in the fact that it included a reasonably representative view of Māori experiences (50% of participants were Maori compared to only 16.5% of the New Zealand population) [44]. This is important given that the prevalence of T2DM is higher in Māori than in non-Māori in New Zealand [1, 2] and given that there is increasing awareness of the importance of providing culturally responsive healthcare for indigenous groups with diabetes [45–47]. A possible limitation of this study was that our participant population was derived from two primary care practices in a single region. Therefore, given that diabetes management and care in primary care is highly dependent on and varied based on the provider and the regional District Health Board (DHB), barriers may vary across different practices/DHBs. Accordingly, an avenue for future research could be to explore barriers to T2DM management at a national level, with participants from a broad array of GP practices from across New Zealand.

## 5. Conclusions

In conclusion, this study shows that while the participants in this study experienced many of the same psychosocial barriers as those reported elsewhere, they can also experience barriers directly associated with their hyperglycaemia (e.g., cognitive impairment) which may impact on their ability to remember to take medication, etc. We also identified that financial concerns and a lack of access to locally relevant resources were key barriers for the participants of this study, and these should be explored further in other people with poorly managed T2DM. Accordingly, financial support for people with diabetes and creating more targeted education resources for disease management (including patient education on where/how to access them) may be areas that could be focused on, both in New Zealand and in other countries, particularly those with indigenous population groups.

## Data Availability

Data sharing is not applicable to this article as no datasets were generated or analysed during the current study.

## Abbreviations

T2DM: Type 2 diabetes mellitus
HbA1c: Glycated haemoglobin
GPs: General practitioners
HCP: Healthcare professional
NZE: New Zealand European.

## References

[1] Coppell K. J., Mann J. I., Williams S. M. (2013). Prevalence of diagnosed and undiagnosed diabetes and prediabetes in New Zealand: findings from the 2008/09 Adult Nutrition Survey. *Journal of the New Zealand Medical Association*.

[2] Ministry of Health 2018 Diabetes; Table 28: indicators by gender, Maori and non-Maori. https://www.health.govt.nz/our-work/populations/maori-health/tatau-kahukura-maori-health-statistics/nga-mana-hauora-tutohu-health-status-indicators/diabetes.

[3] McNaughton D. (2013). ‘Diabesity’ down under: overweight and obesity as cultural signifiers for type 2 diabetes mellitus. *Critical Public Health*.

[4] Best Practice Advocacy Centre New Zealand A rising tide of type 2 diabetes in younger people: what can primary care do?. https://bpac.org.nz/2018/diabetes.aspx.

[5] Ministry of Health 2018 Cardiovascular disease risk assessment and management for primary care. https://www.health.govt.nz/publication/cardiovascular-disease-risk-assessment-and-management-primary-care.

[6] Scheen A., Paquot N., Lefebvre P. (2008). United Kingdom Prospective Diabetes Study (UKPDS): 10 years later. *Revue Medicale de Liege*.

[7] Chepulis L., Morison B., Keenan R., Paul R., Lao C., Lawrenson R. (2021). The epidemiology of diabetes in the Waikato region: an analysis of primary care data. *Journal of Primary Health Care*.

[8] de Pablos-Velasco P., Parhofer K. G., Bradley C. (2014). Current level of glycaemic control and its associated factors in patients with type 2 diabetes across Europe: data from the PANORAMA study. *Clinical Endocrinology*.

[9] Pantalone K. M., Hobbs T. M., Wells B. J. (2015). Clinical characteristics, complications, comorbidities and treatment patterns among patients with type 2 diabetes mellitus in a large integrated health system. *BMJ Open Diabetes Research and Care*.

[10] Best Practice Advocacy Centre New Zealand (2015). 2015 Managing patients with type 2 diabetes: from lifestyle to insulin. *Best Practice Journal*.

[11] American Diabetes Association (2018). *Standards of medical care in Diabetes—2018* Abridged for primary care providers. *Clinical diabetes: a publication of the American Diabetes Association*.

[12] Fernandez A., Schillinger D., Warton E. M. (2011). Language barriers, physician-patient language concordance, and glycemic control among insured Latinos with diabetes: the Diabetes Study of Northern California (DISTANCE). *Journal of General Internal Medicine*.

[13] Nam S., Chesla C., Stotts N. A., Janson S. L., Kroon L. (2011). Barriers to diabetes management: patient and provider factors. *Diabetes Research and Clinical Practice*.

[14] Ahmad N. S., Islahudin F., Paraidathathu T. (2014). Factors associated with good glycemic control among patients with type 2 diabetes mellitus. *Journal of Diabetes Investigation*.

[15] McBrien K. A., Naugler C., Ivers N. (2017). Barriers to care in patients with diabetes and poor glycemic control—a cross-sectional survey. *PLoS One*.

[16] Rushforth B., McCrorie C., Glidewell L., Midgley E., Foy R. (2016). Barriers to effective management of type 2 diabetes in primary care: qualitative systematic review. *The British Journal of General Practice*.

[17] Simmons D., Weblemoe T., Voyle J., Leakehe L., Gatland B., Prichard A. (1998). Personal barriers to diabetes care: lessons from a multi-ethnic community in New Zealand. *Diabetic Medicine*.

[18] Simmons D., Lillis S., Swan J., Haar J. (2007). Discordance in perceptions of barriers to diabetes care between patients and primary care and secondary care. *Diabetes Care*.

[19] Janes R., Titchener J., Pere J., Pere R., Senior J. (2013). Understanding barriers to glycaemic control from the patient’s perspective. *Journal of Primary Health Care*.

[20] Pillay J., Armstrong M. J., Butalia S. (2015). Behavioral programs for type 2 diabetes mellitus. *Annals of Internal Medicine*.

[21] Krebs J., Parry-Strong A., Gamble E. (2013). A structured, group-based diabetes self-management education (DSME) programme for people, families and whanau with type 2 diabetes (T2DM) in New Zealand: an observational study. *Primary Care Diabetes*.

[22] Ministry of Health 2015 Living well with diabetes, a plan for people at high risk of of or living with diabetes 2015-2020. https://www.health.govt.nz/system/files/documents/publications/living-well-with-diabetes-oct15.pdf.

[23] Khan H., Lasker S. S., Chowdhury T. A. (2011). Exploring reasons for very poor glycaemic control in patients with type 2 diabetes. *Primary Care Diabetes*.

[24] Peyrot M., Barnett A., Meneghini L., Schumm-Draeger P. M. (2012). Insulin adherence behaviours and barriers in the multinational global attitudes of patients and physicians in insulin therapy study. *Diabetic Medicine*.

[25] Tong W. T., Vethakkan S. R., Ng C. J. (2015). Why do some people with type 2 diabetes who are using insulin have poor glycaemic control? A qualitative study. *BMJ Open*.

[26] Braun V., Clarke V., Hayfield N., Terry G. (2019). Thematic analysis. *Handbook of Research Methods in Health Social Sciences*.

[27] Byrne C., Kurmas N., Burant C. J., Utech A., Steiber A., Julius M. (2017). Cooking classes: a diabetes self-management support intervention enhancing clinical values. *The Diabetes Educator*.

[28] Rawlings A. M., Sharrett A. R., Schneider A. L. (2014). Diabetes in midlife and cognitive change over 20 years. *Annals of Internal Medicine*.

[29] Ravona-Springer R., Heymann A., Schmeidler J. (2014). Trajectories in glycemic control over time are associated with cognitive performance in elderly subjects with type 2 diabetes. *PLoS One*.

[30] Feil D. G., Pearman A., Victor T. (2009). The role of cognitive impairment and caregiver support in diabetes management of older outpatients. *The International Journal of Psychiatry in Medicine*.

[31] Smalls B. L., Walker R. J., Hernandez-Tejada M. A., Davis K. S., Egede L. E., Campbell J. A. (2012). Associations between coping, diabetes knowledge, medication adherence and self-care behaviors in adults with type 2 diabetes. *General Hospital Psychiatry*.

[32] Gonzalez J. S., Tanenbaum M. L., Commissariat P. V. (2016). Psychosocial factors in medication adherence and diabetes self-management: implications for research and practice. *The American Psychologist*.

[33] Kawamura T., Umemura T., Hotta N. (2012). Cognitive impairment in diabetic patients: can diabetic control prevent cognitive decline?. *Journal of Diabetes Investigation*.

[34] Kontopantelis E., Reeves D., Valderas J. M., Campbell S., Doran T. (2013). Recorded quality of primary care for patients with diabetes in England before and after the introduction of a financial incentive scheme: a longitudinal observational study. *BMJ Quality & Safety*.

[35] Lorincz I. S., Lawson B. C., Long J. A. (2013). Provider and patient directed financial incentives to improve care and outcomes for patients with diabetes. *Current Diabetes Reports*.

[36] Diabetes New Zealand (2021) – Resources. https://www.diabetes.org.nz/resources-1.

[37] Diabetes New Zealand (2021) – What is diabetes. https://www.diabetes.org.nz/whatisdiabetes.

[38] Health Navigator New Zealand What is diabetes?. https://www.healthnavigator.org.nz/health-a-z/d/diabetes-overview/.

[39] Diabetes Foundation Aotearoa Our programmes. https://www.diabetesfoundationaotearoa.nz/our-programmes.

[40] Sukala W. R., Page R. A., Rowlands D. S. (2012). Exercise intervention in New Zealand Polynesian peoples with type 2 diabetes: cultural considerations and clinical trial recommendations. *Australasian Medical Journal*.

[41] Simmons D., Voyle J., Rush E., Dear M. (2010). The New Zealand experience in peer support interventions among people with diabetes. *Family Practice*.

[42] Ross Barnett J., Pearce J., Howes P. (2006). 'Help, educate, encourage?': Geographical variations in the provision and utilisation of diabetes education in New Zealand. *Social Science & Medicine*.

[43] Lambrinou E., Hansen T. B., Beulens J. W. (2019). Lifestyle factors, self-management and patient empowerment in diabetes care. *European Journal of Preventive Cardiology*.

[44] Statistics New Zealand (2019) New Zealand’s population reflects growing diversity. https://www.stats.govt.nz/news/new-zealands-population-reflects-growing-diversity#:~:text=The%20next%20largest%20ethnic%20group,from%2011.8%20percent%20in%202013.

[45] Kram F. Improving Maori access to health care: research report: prepared for the Ministry of Health 2014. https://www.moh.govt.nz.

[46] Crowshoe L. L., Henderson R., Jacklin K., Calam B., Walker L., Green M. E. (2019). Educating for equity care framework: addressing social barriers of indigenous patients with type 2 diabetes. *Canadian Family Physician*.

[47] Harris S. B., Tompkins J. W., TeHiwi B. (2017). Call to action: a new path for improving diabetes care for indigenous peoples, a global review. *Diabetes Research and Clinical Practice*.

